# Electrolyzed Water as a Biocide in Premise Water Networks: A Narrative Review and a Conceptual Framework

**DOI:** 10.3390/pathogens15090962

**Published:** 2026-09-09

**Authors:** Tommaso Russo, Andrea D’Orazio, Angelo Baggiani

**Affiliations:** Department of Translational Research and of New Technologies in Medicine and Surgery, University of Pisa, 56126 Pisa, Italy; t.russo6@studenti.unipi.it (T.R.); a.dorazio@studenti.unipi.it (A.D.)

**Keywords:** hypochlorous acid, water safety, biocides, electrolyzed water, chlorine, water electrolysis, waterborne pathogens

## Abstract

Biocide usage in premises plumbing systems requires a multidimensional evaluation to ensure that all interfering factors are controlled and the microorganism presence is strictly limited or eradicated. Current research on electrolyzed water as a primary water biocide has been limited to isolated aspects of its application, providing an insufficient overview of both biocide properties and usage. Following a narrative literature review, this study proposes a unified framework for analyzing water biocides, structuring our research into a multidimensional approach starting from chemical properties and concluding with implementation challenges. Although electrolyzed water constitutes a robust strategy for water safety, further research is necessary regarding efficacy against amoebae and fungi. Careful consideration must also be given to technical aspects, such as material compatibility and overall persistence.

## 1. Introduction

Water safety has been a major public health achievement, reshaping both the quality of life and the epidemiological background of societies across the world. Biocides have retained their importance in controlling microorganisms in water premises (i.e., building-level plumbing systems downstream of the water utility connection, encompassing both domestic and non-domestic/institutional installations), though developments in these fields remain, to date, fragmented and interspersed. The need for a narrative review has emerged, owing to major legislation changes—including the EU Biocidal Products Regulation (BPR), the recast Drinking Water Directive (EU) 2020/2184, and updated national guidance on *Legionella* prevention—and growing public interest in water and environmental safety.

Chlorine compounds have always maintained a pivotal role amongst water biocides: their action against various microorganisms and their low production cost account for their success. However, powerful disinfection capabilities are usually followed by stronger oxidative activity and overall incompatibility with pipe materials and water infrastructure. Also, environmental pollution must be taken into account: chlorine can persist in water drains, potentially contaminating ecosystems. The need for safer, yet powerful, water biocides stimulated research into electrolyzed water and its main active component: hypochlorous acid.

Its capabilities are broad, even in sectors unrelated to water safety per se [1,2]. Considerable efforts have been made, but the literature lacks a comprehensive review of its action in water systems. Literature is also lacking a common framework for analyzing and implementing biocide usage into water premises: original articles usually retain a limited focus, while implementation in water premises requires a multidimensional and cross-sectional evaluation.

This review proposes a basic framework of biocide analysis for water safety, following a common scheme that links chemistry with biocidal action and practical considerations. To this end, the relevant literature was reviewed and synthesized. The work is divided into (1) chemistry, (2) mechanism of action, and (3) biocidal action sections, prompting a discussion of the major issues in its (4) implementation and some (5) technical challenges, adapting the comparative approach used by Loret et al. [3], who evaluated disinfectant performance against biofilm, protozoa, and *Legionella*. Here, the review extends that comparative logic specifically to electrolyzed water, integrating chemical, mechanistic, and technical dimensions into a single evaluative framework, as detailed [4], though this conceptual framework proved useful not only toward our efforts, but also in giving an overview on hypochlorous acid, outlining the potential applicability of hypochlorous acid to modern water premises—although, as discussed in Section 5, direct evidence from full-scale premise plumbing systems (as opposed to food industry or laboratory settings) remains limited.

### Search Strategy

Relevant literature was identified through searches of PubMed/MEDLINE, Scopus, and Web of Science, covering publications up to June 2026. Search terms included combinations of “electrolyzed water,” “hypochlorous acid,” “chlorine biocide,” “premise plumbing,” “waterborne pathogens,” “biofilm disinfection,” and “disinfection byproducts.” Reference lists of retrieved articles were also screened for additional relevant studies. Given the narrative nature of this review, no formal systematic screening protocol (e.g., PRISMA) was applied; articles were selected and synthesized based on relevance to the proposed conceptual framework, prioritizing studies addressing premise water networks and drinking water applications when available.

## 2. Chlorine Chemistry

Chlorine compounds represent one of the major classes of biocides. Far from being a homogeneous class, they represent a diverse group of molecules with different capabilities and downsides. Concerning water safety, chlorine compounds can be divided into three major groups: (a) hypochlorite-based biocides, which represent the most common choice and include household bleach and hypochlorous acid, (b) stable-chlorine compounds, such as chlorine dioxide, and (c) chloramines, both a byproduct of disinfection and, as monochloramine, a disinfectant per se. Both groups (a) and (c) share a common chemical background, while group (b) stands alone both in chemical properties and actions. The following sections lay the groundwork for this shared foundation, setting aside the chemistry of chlorine dioxide.

Electrolyzed water (EW) is a chlorine-based biocide generated in situ by the electrolysis of a dilute saline (NaCl) solution; its principal active species is hypochlorous acid (HOCl). EW therefore belongs to the same broad family of chlorine disinfectants discussed above, differing chiefly in its on-site mode of production, pH control, and reduced need for chemical storage, rather than in the fundamental chemistry of its active chlorine species (see Section 2, below, on the equilibrium between HOCl and OCl^−^).

Three chemical properties appeared crucial in summarizing both the biocide activity and its reactivity: pH, reduction potential, and lipophilicity. These parameters represent a key summary of biocidal activity and prove a direct correlation between chemical structure and disinfection capabilities. However, they do not act in isolation; these properties cross-influence each other and are strongly susceptible to environmental variables, notably temperature fluctuations and solute composition.

### 2.1. pH Influence

Hypochlorous acid behaves as a weak acid [4], following the subsequent dissociation equation (pKa ≈ 7.5 at 25 °C) described in Equation (1):

(1) $$HOCl⇌{OCl}^{-}+H^{+}$$

**Figure 1:**
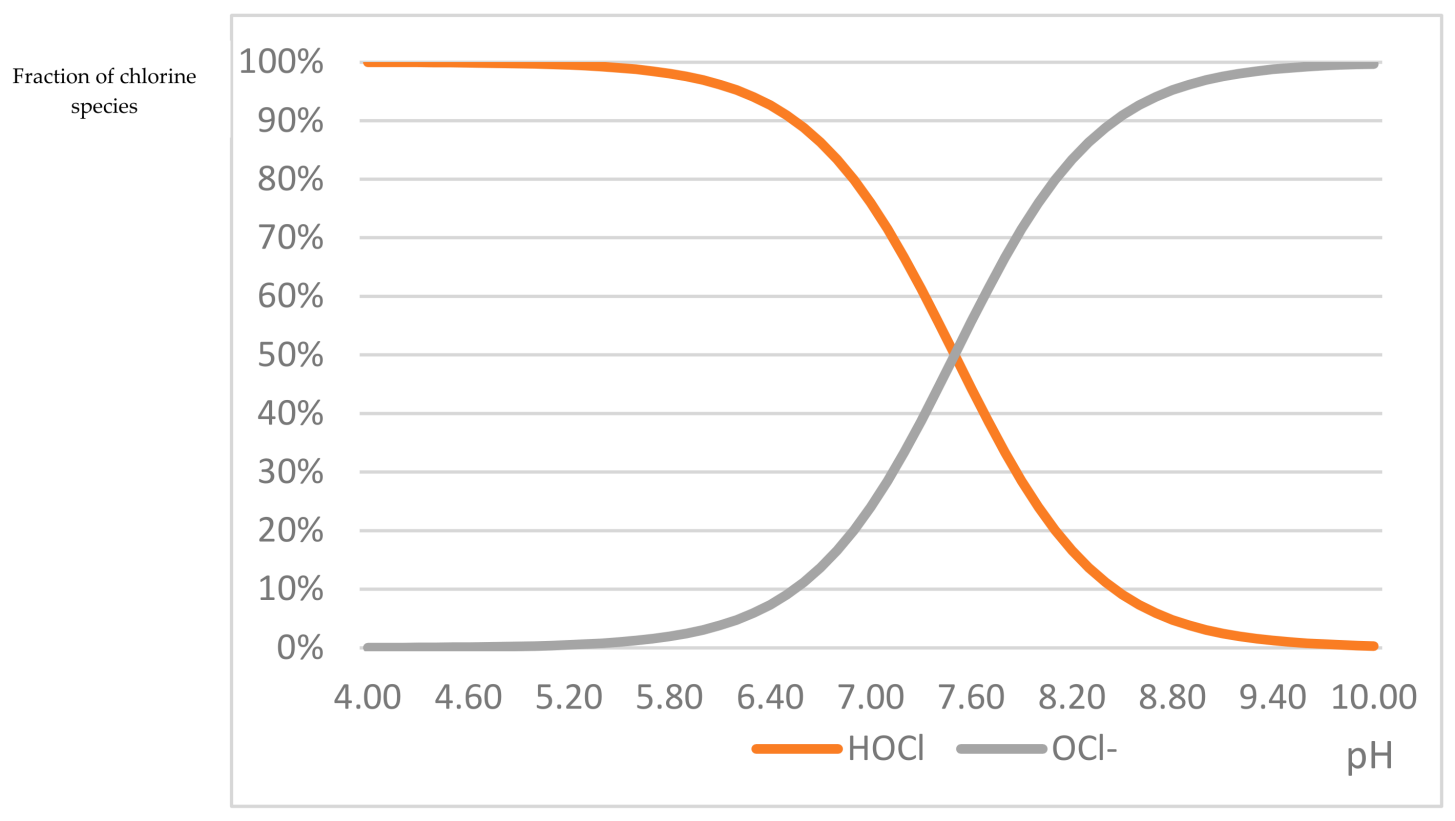
HOCl and OCl^−^ dissociation curve.

This strongly links the action of chlorine compounds to the overall pH. At neutral pH (6.5–7.5), HOCl decomposes at a high rate, owing to a short shelf life; on the other hand, hypochlorite remains stable at alkaline pH (see Technical Challenges). Below pH 6.5, HOCl prevails, while the dissociated base ${OCl}^{-}$ prevails at pH above 7.5, as shown in the dissociation curve (Figure 1). Ultimately, pH determines which chemical species of hypochlorous acid is formed in the solution: as we will see later, HOCl and ${OCl}^{-}$ show relevant differences in action on biomolecules and cells.

It is also possible, at low pH and alongside the presence of free chlorine ions, to also produce chlorine (Cl_2_), which is scarcely soluble in water, so it escapes as free chlorine gas, as per Equation (2).

(2) $$HOCl+H^{+}+{Cl}^{-}\;⇌\;{Cl}_{2}\;\left(g\right)+H_{2}O$$

Formation of chlorine gas without chlorine ions is not possible per se; however, common bleach solutions are contaminated with sodium chloride (NaCl), a byproduct of sodium hypochlorite production. Chlorine gas production starts at a variable pH, depending on both H^+^ and Cl^−^ concentrations, but it never occurs over 4.5 pH [5]. Gaseous chlorine (Cl_2_) possesses limited interaction with most biomolecules; on the other side, the chlorine species within the hypochlorite ion possesses a strong oxidant capability, acting as a strong electrophile (Cl^+^). Thus, the formation of chlorine gas is regarded as ineffective for disinfection usage in water, besides having scarce solubility and being harmful to humans. However, free chlorine (or free residual chlorine) in water is traditionally represented by these three products: hypochlorous acid (HOCl), hypochlorite ion (${OCl}^{-}$), and, in an often-negligible part, chlorine gas (Cl_2_).

### 2.2. Reduction Potential Influence (ORP)

Free chlorine is highly reactive with most biomolecules. Reactions with nitrogen (both in free ammonia form and N-organic compounds, see Equation (3)) produce chloramines; in laboratory measurement, these compounds constitute combined chlorine, and altogether with free chlorine, they constitute a fraction of total chlorine. Chloramines represent a byproduct of chlorine disinfection and produce a typical smell in chlorinated waters. Among these compounds, only monochloramine is used as a biocide in premise water.

(3) $$NH_{3}+HOCl\;\to \;NH_{2}Cl+H_{2}O$$

Reduction potential is a quantitative indicator of oxidizing capabilities: it is safe to assume that a higher potential means better reactivity with biomolecules (see Mechanism of Action). Reduction potential of HOCl is approximately 1.7-fold that of the hypochlorite ion, while monochloramine, even in acidic environment, remains lower than HOCl [6] (Equations (4)–(6)).

(4) $$HOCl+H^{+}+2\;e^{-}\;\to \;{Cl}^{-}+H_{2}O\;\;\;\;\;\;\;\;\;\;\;\;\;\;\;\;\;\;\;\;\;\;\;\;\;\;\;\;\;\;\;\;\;\;\;\;\;\;\;\;\;\;E_{h}^{0}=1.49\;V$$

(5) $$ClO^{-}+H_{2}O+2\;e^{-}\;\to \;{Cl}^{-}+2\;OH-\;\;\;\;\;\;\;\;\;\;\;\;\;\;\;\;\;\;\;\;\;\;\;\;\;\;\;\;\;\;\;\;\;\;E_{h}^{0}=0.9\;V$$

(6) $${NH}_{2}Cl+2H_{2}O+4\;e^{-}\;\to \;{2Cl}^{-}+NH_{3}+2OH^{-}\;\;\;\;\;\;\;\;\;\;\;\;\;\;\;\;\;E_{h}^{0}=0.75\;V$$

Reduction potential is usually measured in standard conditions, while temperature and pH can both influence it deeply, as expressed by Nernst equation. Reduction potential influence at various pH levels is usually represented by *E_h_-pH* diagrams. However, Nernst equation for hypochlorous acid shows that maximum reduction potential appears at lower pH, while higher pH slowly decreases its value and reactivity, beside favoring its dissociation.

Reduction potential may also be an indicator of corrosion, proving useful as a correlation between biocide’s chemical properties and compatibility with pipe materials.

It should be emphasized that ORP could correlate with organic molecule reactivity and, thus, antimicrobial efficacy, but is nonetheless influenced by environmental conditions (see Nernst equation, above) and by the presence of interfering solutes.

### 2.3. Lipophilicity Influence

Lipophilicity has minor influences on reactivity with biomolecules. However, it is of great importance in understanding which sites can be reached by the compound. Proteins and, on a larger scale, cells themselves are usually comprised of both hydrophilic and lipophilic areas: deep penetration into a living microorganism is mediated by lipophilicity.

Evaluating lipophilicity from a quantitative point of view may be challenging in small and unstable molecules (e.g., hypochlorous acid). Standard approaches include both net charge and dipole moments: while the former quality may be easily determined and analyzed, dipole moment is difficult to evaluate. Comparing our main molecules, both HOCl and NH_2_Cl are neutral in net charge, while ${OCl}^{-}$ has a negative charge. Thus, ${OCl}^{-}$ is more prone to diffusion in aqueous solution compared to both hypochlorous acid and monochloramine.

Comparing lipophilicity HOCl and NH_2_Cl may be more difficult for a quantitative analysis. Little to no data on dipole moments is reviewed in literature; also, dipole moment may be of more obscure interpretation in these contexts. Partition coefficient (between water and 1-octanol) directly represents diffusion into both aqueous and lipidic media, mimicking diffusion through plasma membrane. Experimental determination of partition coefficients proved to be inconclusive for HOCl, while computational method appears to be more straightforward for such comparisons. Partition coefficients are usually expressed in logarithmic units ($\log K_{ow}$) with negative values representing affinity for water, while positive values represent more affinity for lipidic media. Also, hydrophilic-lipophilic balance (HLB) represents a valid global metric, ranging from a scale of 0 to 20 with 0 representing maximum lipophilicity and 20 representing maximum hydrophilicity. Using the Chemicalize platform [7], HLB values were estimated at 12.329 for monochloramine and 7.933 for neutral HOCl, with corresponding $\log K_{ow}$ values of 0.025 and 0.318, respectively. While monochloramine tends toward hydrophilicity, HOCl maintains its value around a grey zone, prompting almost-amphoteric properties. Consequently, a more efficient penetration can be expected for HOCl, as demonstrated also by Fukuzaki [4].

It should be noted that the HLB and logKow values reported above are computational estimates (obtained via the Chemicalize platform) rather than experimentally measured quantities; as such, they should be interpreted as indicative of relative trends rather than as definitive, experimentally validated partition coefficients.

HOCl is thus theoretically capable of deeper penetration into cellular membranes, owing to more reactivity with internal components of the cell [4]. On the other hand, hypochlorite ions tend to react with extracellular matrix and external components of the cell wall. Research in this area may prove conclusive in evaluating new biocides and comparing them with existing ones, although designing suitable experimental settings remains challenging. However, no in vivo experimentation has been carried out to date.

### 2.4. From Chemical Properties to Biocidal Action

Any biocide can be considered both in its kinetic and in its dynamic properties: the first explains how fast a particular molecule can act; the second explains the inner interaction between the molecules and the living organisms. Reduction potential and pH constitute the main parameters influencing the dynamic of hypochlorous acid action: pH is essential in determining the species, should it be hypochlorous acid or its hypochlorite ion. Reduction potential, on the other hand, determines the capability of oxidizing and reacting with organic molecules and can be correlated with disinfection power.

Most literature examines just one or two of these properties, focusing on gross reactivity with biomolecules and on CT values (concentration x time product). However, this inner correlation appears pivotal both for a comprehensive research framework and for evaluating molecules for their practical usage.

Lipophilicity, thus, can be regarded as a factor determining the timing and possibility of contact with organic molecules: assuming the cells, as enveloped in hydrophobic lipid bilayer (the plasma membrane), more lipophilic biocides are capable of deep penetration into cytoplasm, while more hydrophilic ones will be confined in extracellular space. A lipophilic biocide will reach deeper structures in less time than hydrophilic molecules, leading to faster disinfection. Also, lipophilicity interacts with pH: partition coefficient can be replaced by apparent partition coefficient (logD), which adjusts partition coefficient to every pH. Although relevant, this has rarely been considered in chlorine compound properties. Analyzing lipophilicity may prove to impact both efficacy and biocide penetration, while outlining a safer profile regarding material compatibility. Although no studies have yet addressed this matter, it represents a promising avenue for future research.

Active chlorine concentration (ACC) can also be considered: in fact, it has been investigated in literature, mostly relating it with efficacy. However, active chlorine concentration retains its role mostly in monitoring and comparison rather than as an underlying factor. It is deeply influenced by both pH and temperature; also, it has no direct correlation to one chlorine species or another: it just represents a common “chlorine equivalent.” Therefore, it has not been considered in general properties of chlorine-based biocides.

It is possible to conclude that a hypochlorite ion exhibits minor reactivity and slower kinetics, being confined to the extracellular environment, while hypochlorous acid tends to be highly reactive and to diffuse deeply into the cells. These three parameters should not be taken as independent variables: pH influences both reduction potential and the prevalence of hypochlorite over hypochlorous acid. It is also safe to assume that drinking water usually maintains a relatively narrow range of pH; temperature, on the other hand, varies greatly between premise plumbing systems.

While reduction potential and lipophilicity determine their kinetics and dynamics, pH determines which species is predominant at a set temperature. Also, temperature and pH influences reduction potential (see Nernst equation), setting a common interdependence of these variables on biocide dynamic and kinetics. It is possible to frame other chlorine biocides into this common chemical framework, exposing common ground for comparison and evaluation. Table 1 summarizes key chemical proprieties of selected chlorine species.

## 3. Electrolyzed Water (EW) Classification

Hypochlorous acid is produced through water electrolysis: a brine solution is used as a substrate, with NaCl in range varying from 0.2% to 6% [2]. Two separate solutions are produced, respectively, at the anode and at the cathode. At the anode (positive terminal), chloride oxidation generates chlorine _2_, HOCl) and protons (H^+^), yielding a low-pH solution (AEW or acid electrolyzed water, pH 2.0–2.7); at the cathode (negative terminal), water reduction generates H_2_ and OH^−^, while Na^+^ cations migrate toward the cathode, yielding a high-pH solution (BEW or basic electrolyzed water, pH 10–13). A mixture of AEW and BEW in different qualities produced solutions of varying pH: weak acidic electrolyzed water (WAEW, pH 5.0–6.5) and neutral electrolyzed water (NEW) can also be produced. BEW has stronger cleaning properties, while NEW is generally preferred for disinfection in premise plumbing, reflecting practical considerations—material compatibility, reduced off-gassing, and favorable persistence at neutral pH—rather than superior chlorine speciation per se [8,9].

Hypochlorous acid is not the sole component of these solutions: there are indeed a variety of radical byproducts and molecules being represented in both AEW and BEW. These species, however, remain largely uncharacterized in current literature, but may presumably pertain to free chlorine and oxygen species, enhancing oxidative stress and reactivity toward biomolecules. Consequently, a comprehensive characterization represents a major gap in EW research, which could prompt the development of new biocides or lead to a better understanding of EW action on living organisms.

As shown in Section 2.1, Cl_2_ gas formation is confined to strongly acidic conditions (pH below approximately 4.5) and is therefore not represented in Figure 1, which spans pH 4–10. Within the pH range relevant to premise water networks, acidification favors the more reactive HOCl species, while alkalinisation shifts speciation toward the less reactive OCl^−^ ion (Figure 1); the HOCl fraction, and thus the fastest disinfection kinetics, in fact peaks in the weakly acidic range (pH 5.0–6.5, WAEW/SAEW). Both AEW and BEW exert a strong corrosion on materials, rendering their application unpractical for most water system. NEW, on the other side, maintains a neutral pH, an unusual feature among other chlorine-based compounds (which are usually set at an alkaline pH).

It is important to emphasize that HOCl and OCl^−^ are not two distinct biocidal agents, but a single conjugate acid-base pair in rapid equilibrium (Equation (1), above). Consequently, at a given bulk-water pH and free available chlorine concentration, the speciation—and hence the theoretical reactivity—of chlorine delivered as electrolytically generated NEW is expected to be equivalent to that of chlorine dosed as sodium hypochlorite at the same pH. Any practical advantage claimed for on-site electrolytic generation over conventional hypochlorite dosing (see Section 7.1, Section 7.3 and Section 7.4) should therefore be attributed not to a fundamentally different chlorine species, but to secondary factors specific to the production and delivery pathway: avoidance of chlorate accumulation in degraded hypochlorite stock, absence of caustic carryover, and associated pH excursions at the injection point, lower ionic/sodium loading, and on-site production logistics that reduce transport and storage requirements.

## 4. Mechanisms of HOCl Antimicrobial and Oxidative Action

Hypochlorous acid (HOCl) reacts with multiple targets both inside and outside cells. Current literature demonstrates its reactivity toward various biomolecules, which can be summarized as follows:

• Formation of chloramines (via reaction with amino acids).
• Oxidation of macromolecules (through reactions with proteins, lipids, and sugars).
• Oxidative stress involving both (a) the depletion of antioxidants and (b) Fenton reactions triggered by heme- and iron-containing groups (e.g., cytochromes and iron-sulfur clusters in bacteria; heme groups in the mitochondria of eukaryotic cells and protozoa).

These mechanisms do not operate as parallel, isolated pathways; instead, their actions frequently intersect. The oxidative stress induced by chlorine compounds is now recognized as a distinct phenomenon in literature, highlighting the role of halogenative stress in human health [6,10]. While these effects depend mainly on OR, both pH and lipophilicity appear determinant in biocide reactions. Together, they ensure HOCl is generated in its active species and successfully diffuses across plasma membrane.

### 4.1. Endogenous Production

The endogenous production of HOCl is widely recognized as the primary microbiocidal pathway of neutrophils, with significant implications for systemic inflammation. HOCl is naturally produced by specific class of heme peroxidases, of which there are four main types in the human body: myeloperoxidase (MPO), eosinophil peroxidase (EPO), lactoperoxidase, and thyroperoxidase. Among these, only MPO can produce hypochlorous acid in significant quantities [8]. MPO is one of the primary enzymes responsible for depleting H_2_O_2_, which serves to regenerate its active site. Consequently, HOCl is a major neutrophil byproduct, exerting its function during the oxidative burst and the physiological inflammatory response.

MPO features an active site operating via two distinct catalytic pathways: a halogenating cycle and a peroxidative cycle [11,12]. The former directly produces HOCl, while the latter regenerates the active site using electron donors such as urate, ascorbate, or various amino acids.

### 4.2. Chloramines

Chloramines represent a broad class of compounds; they include not only inorganic monochloramine, dichloramine, and trichloramine, but also byproducts formed between chlorine compounds and amino acids [9]. Amino groups undergo strong oxidation by HOCl, transforming their amino terminal into -NCl_2_ or -NHCl residues. While these groups are preferential targets for HOCl, they remain unstable, triggering multiple chain reactions that significantly increase intracellular oxidative stress [13].

Another primary target of HOCl is thiol residues -SH [8,14,15], such as those found in cysteine. This reaction produces thiol radicals and consumes GSH (reduced glutathione), one of the cell’s most prominent antioxidant defenses.

### 4.3. Macromolecules Oxidation

Hypochlorous acid is capable of oxidizing major biomolecules within cells, reacting predominantly with thiol groups, double bonds, and nitrogen-containing moieties. Reactivity with biomolecules is determined by both chemical structure and steric accessibility; for instance, nucleic acids hinder HOCl reactivity largely through steric shielding mechanisms [8].

Proteins and carbohydrates undergo a complex process of oxidation [8,11], usually at nitrogenous functional groups; pure homonuclear bonds (e.g., -C-C-) are non-reactive to hypochlorous acid under normal conditions. Protein degradation severely alters cellular functions and enzymatic activity, though different proteins may exhibit varying rates of oxidation [9,12]. Aggregation and side chain modification may also occur, altering protein folding and functioning. On the other hand, nitrogen-rich sugars are more susceptible to hypochlorous acid, leading to a stronger impact on the cell walls of many pathogens. Direct oxidation of macromolecules represents perhaps the most extensive mechanism of action, capable of dismantling cellular machinery on multiple levels.

HOCl is also capable of inactivating various enzymes [16,17], with the notable exception collagenases and other neutrophil enzymes, which are, conversely, activated by HOCl. This activation, coupled with protein lysis, can inhibit protein synthesis within the cell [18].

Lipids are selectively susceptible to degradation: saturated lipids are non-reactive [19], while double bonds can be oxidized by HOCl to generate isomers of chlorohydrin (which are highly unstable). An indirect increase in lipid peroxidation is also possible through radicals produced during the peroxidative cycle of MPO [20]. HOCl can also react with the hydrophilic heads of membrane lipids, leading to increased permeability [21]; specifically, nitrogen groups are more reactive, while phosphate groups show no reactivity. Cholesterol may also be oxidized, producing both chlorohydrins and epoxides without generating free radicals [22].

### 4.4. Oxidative Stress

Hypochlorous acid exerts a peculiar action on the heme groups within proteins. Studies show an increase in intracellular iron following HOCl exposure, leading to an indirect increase in oxidative stress. Iron release is dependent on the reaction with the heme group, which is present in the mitochondria of many aerobic organisms.

Heme degradation occurs naturally through both enzymatic and non-enzymatic pathways. Non-enzymatic pathways depend on free radicals, primarily H_2_O_2_. Literature shows that HOCl can mimic this action [23,24]. Hypochlorous acid is capable of oxidizing two different targets in heme proteins: the ferric center can be oxidized to Fe-OO [25], disabling respiratory and catalytic functions, while bonds can be cleaved within the tetrapyrrolic ring. This cleavage produces various subproducts and impairs iron retention within the heme ring [26,27]. This, in turn, releases free iron into the cell and triggers the Fenton reaction, increasing oxidative stress and, indirectly, non-enzymatic heme degradation. This creates a continuous cycle capable of increasing both intracellular and extracellular oxidative stress and free radical generation.

HOCl ability to interact directly with heme group reveals interesting feedback between hypochlorous acid and MPO. Peroxidase commonly uses heme group in their active site, so HOCl is capable of interacting and dismantling its own enzyme. Heme group in MPO is, in fact, vulnerable to HOCl oxidation, but to a lesser extent owing to its covalent bond [28], though it is possible and documented that HOCl can inhibit this enzyme [26,29].

DNA interactions present a complex landscape; while hypochlorous acid does not show direct reactivity toward nucleic acids (only toward isolated nucleotides or experimentally denatured DNA), DNA damage may be explained by both oxidative stress and histone interactions, leading to the denaturation of both bacterial and eukaryotic genomes. Owing to steric interactions, it may interact more easily with cellular RNA, owing to another factor of interference in protein synthesis [30].

## 5. Antimicrobial Efficacy of Electrolyzed Water

Amongst various types of electrolyzed water, NEW has been extensively tested, particularly in the food industry, where it is widely used for the disinfection of washing water. It should be noted that much of the antimicrobial evidence summarized in this section derives from food processing, surface disinfection, or short-term laboratory studies, which differ from premise plumbing systems in water chemistry, contact surfaces, hydraulic conditions, and exposure duration. Extrapolation of these findings to drinking water distribution and building plumbing systems should therefore be considered cautious, pending confirmatory studies conducted under realistic premise water conditions.

### 5.1. Efficacy Against Planktonic Bacteria

Regarding its antibacterial properties, its efficacy has been widely demonstrated even with very short contact times, especially on free-floating bacteria compared to those aggregated within biofilms. Research conducted on minimally processed vegetables has confirmed that washing for just 1–3 min with neutral electrolyzed water at 50 ppm reduces the presence of *Listeria innocua* and *Salmonella* by approximately 1.0–2.0 log, a result comparable to sodium hypochlorite, but with a significantly better safety and residue profile [31]. Similar results were obtained in washing kitchen utensils, where comparing neutral electrolyzed water with sodium hypochlorite solutions showed equal efficacy, drastically reducing the colony concentration of *Escherichia coli*, *Listeria monocytogenes*, *Pseudomonas aeruginosa*, and *Staphylococcus aureus* in just 1 minute of exposure. The efficacy of neutral electrolyzed water was found to be comparable to that of the sodium hypochlorite solution, with the added advantage of a longer shelf life [32]. Multidrug-resistant bacteria have also been tested [33]; researchers conducted in vitro tests using some of the main pathogens responsible for healthcare-associated infections, including methicillin-resistant *Staphylococcus aureus* (MRSA), *Acinetobacter baumannii*, *Pseudomonas aeruginosa*, and *Enterococcus faecalis*, observing a total absence of bacterial colony growth on silicone discs after being exposed to HOCl.

Mezule et al. also report a good efficacy of water electrolysis on *B. subtilis* spores [34], being three times more effective than chlorine compounds alone. However, large-scale applications need to be addressed on this subject.

### 5.2. Efficacy Against OPPPs

Opportunistic premise plumbing pathogens (OPPPs) are of main interest in water safety. This group [35,36] includes both well-known waterborne pathogens as *Legionella* spp. and largely uncharacterised microorganisms, while other human pathogens showed relevant OPPPs’ properties (*P. aeruginosa* and *Mycobacterium avium*, both relevant in water safety). Main OPPPs’ properties include replication in amoebae, an innate resistance to chlorine disinfection and biofilm formation [35].

Laboratory results are particularly promising, showing a good efficacy on both *Legionella* and other OPPPs in electrolysis cells [37] and pool settings [38]. Residual effect, as expected in common premise plumbing usage, remains relevant after electrolysis cell exposure [39]. With regards to large-scale settings, Migliarina e Ferro [40] proved electrolyzed water efficacy in eradicating *Legionella* spp. from hospital water sources, while NEW also showed decrease in VBNC, as remarked by molecular analysis by Bonetta et al. [41].

### 5.3. Efficacy Against Bacteria in Biofilm

Although its efficacy has been studied less extensively than on free-floating bacteria, there are studies documenting the effectiveness of hypochlorous acid even on bacteria within the polysaccharide matrix of a biofilm [42,43,44]. A study was conducted analysing the growth in mature biofilms of *Listeria monocytogenes* on polystyrene and stainless-steel surfaces [45]. The results showed that neutral electrolyzed water is capable of autonomously penetrating the protective matrix, resulting in a significant reduction of the bacterial population. The antimicrobial action varied significantly between strains and surfaces, with greater efficacy observed against biofilms produced on polystyrene compared to those formed on stainless steel [45]. In industrial processes, sodium hypochlorite is the most widely used biocide; however, current public health concerns related to the formation of carcinogenic organochlorine byproducts are driving research toward safer alternatives such as neutral electrolyzed water. Its efficacy was compared alongside sodium hypochlorite against *E. coli* both in the planktonic phase and in biofilms, revealing that, in addition to having a higher safety profile, neutral electrolyzed water has a more potent biocidal effect, causing greater production of reactive oxygen species (ROS) [46].

However, research under real plumbing system conditions remains limited; current literature relies predominantly on controlled experimental settings. Pipes, particularly during prolonged stagnation periods, provide a favorable microenvironment for biofilm deposition and maturation. Consequently, further studies on mature biofilms are needed to evaluate potential differences in biocide efficacy between mature biofilms and younger, laboratory-cultivated ones.

### 5.4. Efficacy Against Viruses

The recent pandemic has made SARS-CoV-2 an element of great interest in disinfection, and from the perspective of viral disinfection, it has been one of the most studied contaminants [47]. Complete decontamination of the surfaces of medicinal plants from SARS-CoV-2 and another virus from the human coronavirus family was achieved. Studies have also been conducted on other viral strains, including human norovirus [48], which is the leading cause of acute viral gastroenteritis worldwide. Transmission occurs via the fecal-oral or vomit-oral route. Neutral electrolyzed water caused a massive reduction in norovirus genome copies after just 1 min of exposure. Exposure to NEW induced an almost complete loss of receptor binding, degradation of the major capsid protein, and increased viral particle aggregation [48].

### 5.5. Efficacy Against Other Microorganisms

Efficacy against fungi has not been extensively studied; there are only a few in vitro studies where the efficacy of electrolyzed water was evaluated on colonies of *Candida albicans* and *Aspergillus fumigatus*, and it was deemed an effective and economical alternative for their elimination [49].

Efficacy against amoebae should be a topic of research owing to usage of water premises: replication inside amoebae represents one of the main reservoirs of *Legionella* and other OPPPs, including bacteria responsible for antibiotic resistance [50,51,52]. Nevertheless, research on protozoans is lacking. Some studies are following this thread, such as Dupuy et al. [53], which co-measure biocide efficacy on both *Legionella* and *Acanthamoeba*. Also, Coulon [54] showed a good log-reduction using hypochlorite (see Section 2 and Section 3).

## 6. Discussion

### 6.1. Resistance

The development of microbial resistance or tolerance to broad-spectrum oxidative biocides, such as hypochlorous acid, is a highly complex process that differs from well-characterized classic antibiotic resistance mechanisms [55,56,57]. The scientific literature indicates that fully heritable, high-level genetic resistance to this class of agents is uncommon, although specific genetic determinants of reduced susceptibility have been described (e.g., RcrB-mediated HOCl resistance in uropathogenic E. coli [58] and HOCl resistance mechanisms in *Candida albicans* [59], discussed below). In most cases, however, microorganisms rely on the phenotypic tolerance and protective strategies described below rather than on classical resistance mechanisms. Instead, in order to survive exposure to strongly oxidizing disinfectants, pathogens deploy a wide array of phenotypic tolerance and protective strategies, owing to the complex and multisystemic action of chlorine biocides onto prokaryotic cells. Casini et al. showed that long-term exposure induces chlorine resistance in *Legionella* [60].

Prominent among these are the complex formation of biofilms, the upregulation of cellular defenses against oxidative stress, the activation of efflux pumps, and alterations in cell envelope permeability [61]. One possibility is cross-resistance between chlorine compounds and antibiotics: biocides target a variety of machinery, thus requiring a complex and multigenic response. This may enhance, as a collateral adaptation, resistance to antibiotics, which is more prone to exponential resistance mechanism: Adefisoye et Olaniran [62] explained possible interaction mechanism between biocide and antibiotic resistance. Casini et al. also demonstrated a correlation between chlorine susceptibility and antibiotic resistance in *A. baumanii* [63].

Though suggestive from a molecular point of view, more research on this point should be addressed.

### 6.2. Biofilm-Mediated Tolerance

Biofilms are a primary mechanism by which microbes survive exposure to both antibiotics and oxidizing agents like hypochlorous acid. The extracellular polymeric matrix limits penetration of reactive species and creates microenvironments with reduced metabolic activity and increased expression of stress response genes [64], thus providing a safe milieu for bacterial persistence after biocide usage. Biofilm cells also exhibit increased rates of horizontal gene transfer, which can disseminate resistance/tolerance determinants among resident bacteria; combined with the physical and physiological protection described below; this contributes to the long-term survival of biofilm communities at disinfectant concentrations that would be lethal to planktonic cells.

When bacteria aggregate on a surface, they secrete a dense matrix of EPS, composed of sugars, proteins, and extracellular DNA.

This massive difference in vulnerability between free-floating and aggregated bacteria was quantified on the bactericidal efficacy of electrolyzed water against *Listeria* [43,64]. Two experimental studies [64,65] demonstrated that while planktonic cells (free-floating) are destroyed by electrolyzed water in a matter of seconds, bacteria protected within the biofilm require minutes of uninterrupted exposure to suffer the same lethal reduction.

The structural reason for this delay was illustrated by Zhang et al. [66]: the EPS matrix acts as a formidable chemical “sacrificial shield.“ When the hypochlorous acid comes in contact with the biofilm, hypochlorous acid and other NEW components react with external polymers, consuming their ORP and exhausting the disinfecting power before it can penetrate deeply [43,67].

This passive barrier becomes even more formidable in multi-species biofilms. As highlighted by Yan et al. [43], alliances between different bacterial families make the matrix much denser and tougher. Confirming these dynamics, Panebianco et al. [45] documented how survival to treatments depends almost entirely on the robustness of this network, which manages to shelter “survivor cells“ hidden at the center of the colony, saving them from acute oxidation.

### 6.3. Oxidative Stress Defense Systems

Microbes exposed to hypochlorous acid upregulate antioxidant enzymes (e.g., catalase and superoxide dismutase), DNA repair pathways, chaperones, and redox-balancing metabolic shifts (e.g., increased NADPH production) [58,68,69,70]. These responses confer tolerance rather than true resistance—allowing survival during exposure but not necessarily growth at higher concentrations [59,71].

### 6.4. Efflux Pumps and Envelope Modifications

Efflux pumps can export toxic compounds, including some oxidizing agents, and are often upregulated under disinfectant stress [72]. Changes in cell membrane permeability or composition further limit entry or reactivity of biocidal agents [60]. Efflux pumps contribute modestly by exporting toxic compounds but are more relevant for quaternary ammonium compounds than for highly reactive oxidants like those found in hypochlorous acid [61].

### 6.5. Co-Selection and Horizontal Gene Transfer

Sublethal exposure to biocides can select for strains with enhanced efflux capacity or stress responses; these traits may be genetically linked with antibiotic resistance determinants on mobile elements (plasmids/transposons), facilitating co-selection [62,73,74]. Biofilms further promote horizontal gene transfer among resident bacteria [75].

### 6.6. VBNC

The most studied survival mechanism in response to the oxidative stress of hypochlorous acid is the induction of the VBNC (viable but non-culturable) state. When bacteria are exposed to hypochlorous acid concentrations insufficient to cause immediate lysis, they enter a sort of metabolic hibernation.

As demonstrated by a major proteomic analysis conducted on *Listeria monocytogenes* [76], the intense oxidative stress caused by the SAEW induces bacteria to alter protein translation and ribosome biosynthesis. The bacterium shrinks, reduces nutrient absorption, and alters the fluidity of its own cell membrane.

The clinical and industrial danger of this state is significant: as highlighted by studies on *Yersinia enterocolitica* [77], in this state, bacteria are invisible to normal laboratory analysis based on culture plates (appearing falsely “killed”), but they maintain genome integrity and can “resuscitate” [78,79], becoming pathogenic again when environmental conditions improve.

The persistence of VBNC cells is of direct practical relevance to premise water safety: because VBNC organisms evade detection by standard culture-based monitoring, their presence can lead to a false sense of security regarding disinfection efficacy, while the cells retain the potential to resuscitate and regain virulence once favorable conditions are restored. This is of particular concern for OPPPs such as *Legionella* spp. in premise plumbing, where intermittent or suboptimal biocide exposure—rather than complete inactivation—is common [80].

Casini et al. [80] proved the persistence of this viable form during long-term monochloramine disinfection, showing OPPPs’ adaptability to sustained chlorine stress. Although no conclusive studies have examined this matter, it can be hypothesized that a monotonous chemical stimulus tends to produce long-lasting and insidious bacterial adaptation. On this matter, we hypothesize that periodic switching between biocides could reduce the risk of sustained phenotypic adaptation, although this remains speculative and unsupported by direct evidence in premise water systems.

## 7. Technical Challenges

Technical challenges are rarely reported extensively in the current literature, and they are more considered as a mere practical knowledge than a subject of scientific interest. Indeed, providing a clear-cut overview of every main issue in using hypochlorous acid and EW may directly influence efficacy and usage, prompting a tailored solution on every water premises. This highlights the need for more comprehensive research on these aspects, integrating efficacy with microbial resistance mechanisms.

### 7.1. Biocidal Persistence

Hypochlorous acid has a short persistence: long-term storage is impractical, so it has to be locally produced. Also, over long-stretching water network, it can be necessary to implement multiple boosting stations across the whole route.

Factors influencing persistence are various [81]: pH, temperature, pipe material, organic matter (less relevant in drinkable water network compared to natural sources), and biofilm. Thus, biological matter can progressively hinder biocidal activity, inactivating it alongside with the mere distance decay: its small shelf life compared to hypochlorite guarantees a better environmental profile, but it can create suboptimal conditions for biocidal action.

EW requires strict monitoring alongside the pipe routes or onto the returning loop circuit, showing effective consumption into the water network. Consumption may vary depending on the season and the overall network conditions: particular attention should also be addressed to water tanks and dead-end pipe sections (“dead legs”), i.e., stagnant zones with little or no flow, where NEW decay may provide suboptimal biocide action.

### 7.2. Production Issues

Electrolyzed water can be produced in both bi-chamber and mono-chamber reactors [40]. Bi-chamber membrane-reactors guarantee better control over byproducts, separating compartments with a diaphragm, but it is more expensive to operate. On the other hand, mono-chamber system retains a better cost over a worse quality profile: having no separation between BEW and AEW, every byproduct is virtually into the EW [82].

Membrane reactors are more prone to fouling due to cationic impurities: strict control on the brine solution inlet is thus recommended and may imply a pre-treatment of water processing. Using salt as an electrolyte may be more corrosive on the electrodes than alternative electrolytes [81].

### 7.3. Disinfection Byproducts (DBP)

Chlorine biocides react with organic matter, producing disinfection byproducts: these compounds remain mostly undiscovered [83]. Trihalomethanes (THM) and haloacetic acids (HAA) are the two major classes, by quantity, in water DBP [65]. Every halogen in water, due to its reactivity, is capable of producing DBP; however, every biocide has its own reactivity, producing different DBP. In contrast with hypochlorite, EW’s DBPs remain largely unexplored. A favorable THM profile relative to hypochlorite might theoretically be expected since THM formation is generally enhanced at alkaline pH, while the near-neutral pH of NEW could contribute to lower THM formation. However, this prediction is not consistently supported by the available evidence: Migliarina and Ferro reported a small increase, rather than a decrease, in THM [40] though major research on DBP is required.

For electrolytically generated chlorine, chlorate (ClO_3_−) and perchlorate (ClO_4_−) represent a primary regulatory concern, arguably of greater relevance than THMs: chlorate accumulates both in aging hypochlorite stock and within electrolysis cells during operation and is capped at 0.25 mg/L under the EU Drinking Water Directive (EU) 2020/2184. Characterization of chlorate/perchlorate formation and accumulation in EW systems should be regarded as a priority area for future research, alongside THM and HAA formation [84,85,86].

On the other hand, HAA production remains unchallenged at lower pH. Nitrogen-based DBPs are more typically produced by monochloramine biocide: in fact, DBPs are a direct product of oxidation of biomolecules, which encounter hydrolysis and dissociate into these compounds [87]. The combined effect of these changes, along with characterization of DBP, remains pivotal in exploring EW and hypochlorous acid compatibility.

Hypochlorous acid’s shorter environmental persistence may plausibly translate into a more favorable DBP safety profile compared to more persistent chlorine compounds; however, given that EW-associated DBPs—including chlorate and perchlorate—remain largely uncharacterized, this claim should currently be regarded as a hypothesis rather than an established conclusion, pending dedicated comparative studies.

### 7.4. Compatibility with Pipe Materials

One major issue in implementing biocides into water system is represented by compatibility with pipe materials. Literature lacks studies correlating EW and pipe corrosion directly; thus, it is possible to deduce some general considerations based on general review. It is extremely difficult to reproduce real water network conditions in laboratory experiments about corrosion and long-term degradation [88]. A higher ORP, by a chemical point of view, may correlate with higher corrosion, acting on pipes in the same way as biofilm and pathogens [66]. As shown by Cullom et al. [89,90], interaction between pipe materials and biocides is complex and dependent on multiple factors. Temperature plays a role, as warmer conditions favor reaction and corrosion, while also stagnation may enhance reaction with pipe materials. Metal pipes (e.g., iron or copper) appear as more prone to corrosion and chlorine reactivity [91,92], while plastic pipes provide a generally safer profile, but with greater variability between various polymers [93,94]. NEW also has a neutral pH, which can hinder cross-reaction with pipe material. Although a comprehensive chemical analysis of chlorine dioxide lies beyond the scope of this review, preliminary evidence based on a single available reference [88] suggests that NEW might also present a less aggressive profile than more reactive species such as chloramines and chlorine dioxide; however, dedicated comparative reviews are required to confirm these observations.

It is of particular note of this discrepancy between commercial experience and research topic: even if pipe compatibility is largely understood by local water managers, it remains a niche water research topic. New frameworks are emerging, such as Cullom [89,90], in which pipe compatibility is taken into account, though more research on the topic is needed to establish a clear comparison between chlorine compounds in pipe compatibility.

## 8. Future Directions

Hypochlorous acid and electrolyzed water represent a modern approach to water safety. Their unique way of production, alongside their peculiar oxidation capabilities, may represent a step forward in ensuring broad water safety. Drinkable water is a fragile milieu, sensitive to major changes in chemical composition: literature lacks a major approach in both its choice and its characterization. We hope to provide a basis for future research.

Among the gaps identified in this review, three should be regarded as priorities for future research: (1) characterization of EW-specific disinfection byproducts, particularly chlorate and perchlorate, under realistic premise-plumbing conditions; (2) standardized, head-to-head studies of pipe material compatibility across EW, hypochlorite, chloramine, and chlorine dioxide; and (3) efficacy and persistence testing conducted directly in full-scale or pilot premise water networks, rather than in food processing or bench-scale laboratory settings, with particular attention to amoebae and fungi.

## 9. Conclusions

Water disinfection has proven to be a major challenge in public health. Chlorine compounds, amid initial enthusiasm, reportedly required more careful practical considerations. Choosing a water biocide is not an easy task: efficacy should be balanced with environmental safety, absence of toxicity, and material compatibility. Many water networks pose complex challenges: pipe material can be unwillingly corroded, while older designs may prove biocide diffusion to be difficult. Careful on-site monitoring and evaluation should be followed, though establishing a common framework for water biocide study appears advisable.

Hypochlorous acid shows peculiar chemical properties, both as the active ingredient of many chlorine compounds and, per se, as a standalone compound. Biocidal action appears deeply intertwined with oxidative stress capability, acting on many cellular machineries at once: this also provides a clue to probable resistance mechanism. Though, it is difficult to achieve complete resistance, as the molecules involved are many and there is a cascade of oxidation pathways. Efficacy against many planktonic microorganisms is well documented, particularly in food industry and laboratory settings, though—as noted in Section 5—evidence specific to drinking water and premise plumbing applications remains comparatively limited, and further research on fungi and protozoa is needed. Some evidence suggests that HOCl may achieve greater biofilm penetration than hypochlorite while maintaining a more favorable safety profile than monochloramine; however, the evidence based on this specific point remains too limited to draw firm conclusions, and further comparative studies are needed. Electrolyzed water provides a safe source of hypochlorous acid: voltage, temperature, pH, chlorine concentration, and ORP should be closely monitored. Chlorine usage in the circuit should be accounted for by measuring chlorine and biocide levels in network endpoints. Although current DBP research offers limited insight into specific aspects of NEW safety, from a chemical standpoint, it may be assumed to be generally safer than most traditional chlorine compounds. Chlorine dioxide—whose chemistry was excluded from the scope of this review (Section 2) but which is briefly mentioned here for context—has considerable disinfection efficacy but has been reported to show poor pipe material compatibility in a single available study; a dedicated comparative review of chlorine dioxide would be needed to substantiate this further.

From a bird’s-eye view, research on electrolyzed water has mainly focused on food applications and its disinfection properties, though a potential for broader studies into water premises is established. Examining a water biocide should not merely be put under the light of biochemical potency, nor under technical issues. Indeed, it should provide a more comprehensive look, owing to transversal and translational approaches.

## Figures and Tables

**Table 1:** Summary comparison of the chemical properties of the main chlorine species discussed in Section 2, operationalizing the pH/reduction potential/lipophilicity framework.

Parameter	HOCl	OCl^−^	NH_2_Cl (Monochloramine)
*Predominant pH range*	<7.5	>7.5	N/A (distinct species)
*Reduction potential (Eh0 vs. SHE)*	1.49 V	0.90 V	0.75 V
*Net charge*	Neutral	Negative (−1)	Neutral
*Relative lipophilicity (estimated HLB)*	7.93 (more lipophilic)	Low (ionic)	12.33 (more hydrophilic)
*Predominant site of action*	Intracellular (membrane penetration)	Extracellular/matrix	Predominantly extracellular

## Data Availability

No new data were created or analyzed in this study. Data sharing is not applicable to this article.

## Abbreviations

The following abbreviations are used in this manuscript:

ACC	Active chlorine concentration
AEW	Acid electrolyzed water
BEW	Basic electrolyzed water
CT	Concentration x time product, measure of biocide effectiveness
DBP	Disinfection byproducts
EW	Electrolyzed water
EPS	Extracellular polymeric substances
HAA	Haloacetic acids
HLB	Hydrophilic-lipophilic balance
NEW	Neutral electrolyzed water
OPPPs	Opportunistic premise plumbing pathogens
ORP	Reduction potential influence
THM	Trihalomethanes
VBNC	Viable but non-culturable
WAEW	Weak acidic electrolyzed water

## References

[1] Hu D., Kabayama S., Watanabe Y., Cui Y. (2024). Health Benefits of Electrolyzed Hydrogen Water: Antioxidant and Anti-Inflammatory Effects in Living Organisms. Antioxidants.

[2] Chen B.-K., Wang C.-K. (2022). Electrolyzed Water and Its Pharmacological Activities: A Mini-Review. Molecules.

[3] Loret J.F., Robert S., Thomas V., Lévi Y., Cooper A.J., McCoy W.F. (2005). Comparison of Disinfectants for Biofilm, Protozoa and Legionella Control. J. Water Health.

[4] Fukuzaki S. (2006). Mechanisms of Actions of Sodium Hypochlorite in Cleaning and Disinfection Processes. Biocontrol Sci..

[5] Len S.-V., Hung Y.-C., Chung D., Anderson J.L., Erickson M.C., Morita K. (2002). Effects of Storage Conditions and pH on Chlorine Loss in Electrolyzed Oxidizing (EO) Water. J. Agric. Food Chem..

[6] Panasenko O.M., Torkhovskaya T.I., Gorudko I.V., Sokolov A.V. (2020). The Role of Halogenative Stress in Atherogenic Modification of Low-Density Lipoproteins. Biochem. Mosc..

[7] ChemAxon Chemicalize Platform for Chemical Property Prediction. https://chemicalize.com.

[8] Panasenko O.M., Gorudko I.V., Sokolov A.V. (2013). Hypochlorous Acid as a Precursor of Free Radicals in Living Systems. Biochem. Mosc..

[9] Hawkins C.L., Pattison D.I., Davies M.J. (2003). Hypochlorite-Induced Oxidation of Amino Acids, Peptides and Proteins. Amino Acids.

[10] Nishikawa T., Miyahara E., Horiuchi M., Izumo K., Okamoto Y., Kawai Y., Kawano Y., Takeuchi T. (2011). Myeloperoxidase-Derived Halogenative Stress, a Possible Cause of Benzene Leukemogenesis. Blood.

[11] Dalle-Donne I., Rossi R., Giustarini D., Gagliano N., Lusini L., Milzani A., Di Simplicio P., Colombo R. (2001). Actin Carbonylation: From a Simple Marker of Protein Oxidation to Relevant Signs of Severe Functional Impairment. Free Radic. Biol. Med..

[12] Davies M.J. (2005). The Oxidative Environment and Protein Damage. Biochim. Biophys. Acta (BBA) Proteins Proteom..

[13] Hawkins C.L., Davies M.J. (1998). Hypochlorite-Induced Damage to Proteins: Formation of Nitrogen-Centred Radicals from Lysine Residues and Their Role in Protein Fragmentation. Biochem. J..

[14] Pullar J.M., Vissers M.C.M., Winterbourn C.C. (2000). Living with a Killer: The Effects of Hypochlorous Acid on Mammalian Cells. IUBMB Life.

[15] Varatnitskaya M., Degrossoli A., Leichert L.I. (2021). Redox Regulation in Host-Pathogen Interactions: Thiol Switches and Beyond. Biol. Chem..

[16] Hawkins C.L., Davies M.J. (2005). Inactivation of Protease Inhibitors and Lysozyme by Hypochlorous Acid:  Role of Side-Chain Oxidation and Protein Unfolding in Loss of Biological Function. Chem. Res. Toxicol..

[17] Siddiqui T., Zia M.K., Ali S.S., Ahsan H., Khan F.H. (2018). Insight into the Interactions of Proteinase Inhibitor- Alpha-2-Macroglobulin with Hypochlorite. Int. J. Biol. Macromol..

[18] McKenna S.M., Davies K.J. (1988). The Inhibition of Bacterial Growth by Hypochlorous Acid. Possible Role in the Bactericidal Activity of Phagocytes. Biochem. J..

[19] Andrés C.M.C., Pérez de la Lastra J.M., Juan C.A., Plou F.J., Pérez-Lebeña E. (2022). Hypochlorous Acid Chemistry in Mammalian Cells—Influence on Infection and Role in Various Pathologies. Int. J. Mol. Sci..

[20] Niki E. (2009). Lipid Peroxidation: Physiological Levels and Dual Biological Effects. Free Radic. Biol. Med..

[21] Vissers C.M.M., Carr C.A., Chapman L.P.A. (1998). Comparison of Human Red Cell Lysis by Hypochlorous and Hypobromous Acids: Insights into the Mechanism of Lysis. Biochem. J..

[22] van den Berg J., Winterbourn C., Kuypers F. (1993). Hypochlorous Acid-Mediated Modification of Cholesterol and Phospholipid: Analysis of Reaction Products by Gas Chromatography-Mass Spectrometry. J. Lipid Res..

[23] Abu-Soud H.M., Maitra D., Shaeib F., Khan S.N., Byun J., Abdulhamid I., Yang Z., Saed G.M., Diamond M.P., Andreana P.R. (2014). Disruption of Heme-Peptide Covalent Cross-Linking in Mammalian Peroxidases by Hypochlorous Acid. J. Inorg. Biochem..

[24] Souza C.E.A., Maitra D., Saed G.M., Diamond M.P., Moura A.A., Pennathur S., Abu-Soud H.M. (2011). Hypochlorous Acid-Induced Heme Degradation from Lactoperoxidase as a Novel Mechanism of Free Iron Release and Tissue Injury in Inflammatory Diseases. PLoS ONE.

[25] Candeias L.P., Stratford M.R., Wardman P. (1994). Formation of Hydroxyl Radicals on Reaction of Hypochlorous Acid with Ferrocyanide, a Model Iron(II) Complex. Free Radic. Res..

[26] Maitra D., Byun J., Andreana P.R., Abdulhamid I., Saed G.M., Diamond M.P., Pennathur S., Abu-Soud H.M. (2011). Mechanism of Hypochlorous Acid Mediated Heme Destruction and Free Iron Release. Free Radic. Biol. Med..

[27] Maitra D., Byun J., Andreana P.R., Abdulhamid I., Diamond M.P., Saed G.M., Pennathur S., Abu-Soud H.M. (2011). Reaction of Hemoglobin with HOCl: Mechanism of Heme Destruction and Free Iron Release. Free Radic. Biol. Med..

[28] Furtmüller P.G., Zederbauer M., Jantschko W., Helm J., Bogner M., Jakopitsch C., Obinger C. (2006). Active Site Structure and Catalytic Mechanisms of Human Peroxidases. Arch. Biochem. Biophys..

[29] Maitra D., Shaeib F., Abdulhamid I., Abdulridha R.M., Saed G.M., Diamond M.P., Pennathur S., Abu-Soud H.M. (2013). Myeloperoxidase Acts as a Source of Free Iron during Steady-State Catalysis by a Feedback Inhibitory Pathway. Free. Radic. Biol. Med..

[30] Stanley N.R., Pattison D.I., Hawkins C.L. (2010). Ability of Hypochlorous Acid and N-Chloramines to Chlorinate DNA and Its Constituents. Chem. Res. Toxicol..

[31] Abadias M., Usall J., Oliveira M., Alegre I., Viñas I. (2008). Efficacy of Neutral Electrolyzed Water (NEW) for Reducing Microbial Contamination on Minimally-Processed Vegetables. Int. J. Food Microbiol..

[32] Deza M.A., Araujo M., Garrido M.J. (2007). Efficacy of Neutral Electrolyzed Water to Inactivate *Escherichia coli*, *Listeria monocytogenes*, *Pseudomonas aeruginosa*, and *Staphylococcus aureus* on Plastic and Wooden Kitchen Cutting Boards. J. Food Prot..

[33] Palau M., Muñoz E., Lujan E., Larrosa N., Gomis X., Márquez E., Len O., Almirante B., Abellà J., Colominas S. (2022). In Vitro and In Vivo Antimicrobial Activity of Hypochlorous Acid against Drug-Resistant and Biofilm-Producing Strains. Microbiol. Spectr..

[34] Mezule L., Reimanis M., Krumplevska V., Ozolins J., Juhna T. (2013). Comparing Electrochemical Disinfection with Chlorination for Inactivation of Bacterial Spores in Drinking Water. Water Supply.

[35] Falkinham J.O. (2015). Common Features of Opportunistic Premise Plumbing Pathogens. Int. J. Environ. Res. Public Health.

[36] Falkinham J.O., Hilborn E.D., Arduino M.J., Pruden A., Edwards M.A. (2015). Epidemiology and Ecology of Opportunistic Premise Plumbing Pathogens: *Legionella pneumophila*, *Mycobacterium avium*, and *Pseudomonas aeruginosa*. Environ. Health Perspect..

[37] Cossali G., Routledge E.J., Ratcliffe M.S., Blakes H., Fielder J.E., Karayiannis T.G. (2016). Inactivation of *E. coli*, *Legionella* and *Pseudomonas* in Tap Water Using Electrochemical Disinfection. J. Environ. Eng..

[38] Nakajima N., Nakano T., Harada F., Taniguchi H., Yokoyama I., Hirose J., Daikoku E., Sano K. (2004). Evaluation of Disinfective Potential of Reactivated Free Chlorine in Pooled Tap Water by Electrolysis. J. Microbiol. Methods.

[39] Delaedt Y., Daneels A., Declerck P., Behets J., Ryckeboer J., Peters E., Ollevier F. (2008). The Impact of Electrochemical Disinfection on *Escherichia Coli* and *Legionella pneumophila* in Tap Water. Microbiol. Res..

[40] Migliarina F., Ferro S. (2014). A Modern Approach to Disinfection, as Old as the Evolution of Vertebrates. Healthcare.

[41] Bonetta S., Pignata C., Bonetta S., Meucci L., Giacosa D., Marino E., Gorrasi I., Gilli G., Carraro E. (2018). Effectiveness of a Neutral Electrolysed Oxidising Water (NEOW) Device in Reducing *Legionella pneumophila* in a Water Distribution System: A Comparison between Culture, qPCR and PMA-qPCR Detection Methods. Chemosphere.

[42] Aherne O., Ortiz R., Fazli M.M., Davies J.R. (2022). Effects of Stabilized Hypochlorous Acid on Oral Biofilm Bacteria. BMC Oral Health.

[43] Yan P., Chelliah R., Jo K., Selvakumar V., Chen X., Jo H., Oh D.H. (2022). Stability and Antibiofilm Efficiency of Slightly Acidic Electrolyzed Water Against Mixed-Species of *Listeria monocytogenes* and *Staphylococcus aureus*. Front. Microbiol..

[44] Lin H., Zhu X., Wang Y., Yu X. (2017). Effect of Sodium Hypochlorite on Typical Biofilms Formed in Drinking Water Distribution Systems. J. Water Health.

[45] Panebianco F., Alvarez-Ordóñez A., Oliveira M., Ferreira S., Lovisolo S., Vono C., Cannizzo F.T., Chiesa F., Civera T., Di Ciccio P. (2025). Effect of Neutral Electrolyzed Water on Biofilm Formed by Meat-Related *Listeria monocytogenes*: Intraspecies Variability and Influence of the Growth Surface Material. Int. J. Food Microbiol..

[46] Meireles A., Ferreira C., Melo L., Simões M. (2017). Comparative Stability and Efficacy of Selected Chlorine-Based Biocides against *Escherichia coli* in Planktonic and Biofilm States. Food Res. Int..

[47] Block M.S., Rowan B.G. (2020). Hypochlorous Acid: A Review. J. Oral Maxillofac. Surg..

[48] Moorman E., Montazeri N., Jaykus L.-A. (2017). Efficacy of Neutral Electrolyzed Water for Inactivation of Human Norovirus. Appl. Environ. Microbiol..

[49] Unal N., Karadağ A., Yanik K., Bilgin K., Gunaydin M., Birinci A. (2014). Analysis of in Vitro Efficiency of Electrolyzed Water Against Fungi Species Frequently Detected in Nosocomial Infections. Univers. J. Microbiol. Res..

[50] Bornier F., Zas E., Potheret D., Laaberki M.-H., Coupat-Goutaland B., Charpentier X. (2021). Environmental Free-Living Amoebae Can Predate on Diverse Antibiotic-Resistant Human Pathogens. Appl. Environ. Microbiol..

[51] Greub G., Raoult D. (2004). Microorganisms Resistant to Free-Living Amoebae. Clin. Microbiol. Rev..

[52] Thomas V., McDonnell G., Denyer S.P., Maillard J.-Y. (2010). Free-Living Amoebae and Their Intracellular Pathogenic Microorganisms: Risks for Water Quality. FEMS Microbiol. Rev..

[53] Dupuy M., Mazoua S., Berne F., Bodet C., Garrec N., Herbelin P., Ménard-Szczebara F., Oberti S., Rodier M.-H., Soreau S. (2011). Efficiency of Water Disinfectants against *Legionella pneumophila* and *Acanthamoeba*. Water Res..

[54] Coulon C., Collignon A., McDonnell G., Thomas V. (2010). Resistance of *Acanthamoeba* Cysts to Disinfection Treatments Used in Health Care Settings. J. Clin. Microbiol..

[55] Baran A., Kwiatkowska A., Potocki L. (2023). Antibiotics and Bacterial Resistance—A Short Story of an Endless Arms Race. Int. J. Mol. Sci..

[56] Blair J.M.A., Webber M.A., Baylay A.J., Ogbolu D.O., Piddock L.J.V. (2015). Molecular Mechanisms of Antibiotic Resistance. Nat. Rev. Microbiol..

[57] Tenover F.C. (2006). Mechanisms of Antimicrobial Resistance in Bacteria. Am. J. Med..

[58] Crompton M.E., Gaessler L.F., Tawiah P.O., Polzer L., Camfield S.K., Jacobson G.D., Naudszus M.K., Johnson C., Meurer K., Bennis M. (2023). Expression of RcrB Confers Resistance to Hypochlorous Acid in Uropathogenic *Escherichia coli*. J. Bacteriol..

[59] Douglas L.M., Min K., Konopka J.B. (2023). *Candida albicans* Resistance to Hypochlorous Acid. mBio.

[60] Casini B., Buzzigoli A., Cristina M.L., Spagnolo A.M., Giudice P.D., Brusaferro S., Poscia A., Moscato U., Valentini P., Baggiani A. (2014). Long-Term Effects of Hospital Water Network Disinfection on Legionella and Other Waterborne Bacteria in an Italian University Hospital. Infect. Control Hosp. Epidemiol..

[61] Maillard J.-Y., Pascoe M. (2024). Disinfectants and Antiseptics: Mechanisms of Action and Resistance. Nat. Rev. Microbiol..

[62] Adefisoye M.A., Olaniran A.O. (2022). Does Chlorination Promote Antimicrobial Resistance in Waterborne Pathogens? Mechanistic Insight into Co-Resistance and Its Implication for Public Health. Antibiotics.

[63] Casini B., Minacori M., Buzzigoli A., Valentini P., Morici P., Barnini S., Tascini C., Menichetti F., Rossolini G., Privitera G. (2011). Is Multi-Drugs Resistant *Acinetobacter baumannii* Epidemic Spread Related to Reduced Susceptibility to Biocides?. BMC Proc..

[64] Hao J., Zhang J., Zheng X., Zhao D. (2022). Bactericidal Efficacy of Slightly Acidic Electrolyzed Water (SAEW) against *Listeria monocytogenes* Planktonic Cells and Biofilm on Food-Contact Surfaces. Food Qual. Saf..

[65] Han Q., Song X., Zhang Z., Fu J., Wang X., Malakar P.K., Liu H., Pan Y., Zhao Y. (2017). Removal of Foodborne Pathogen Biofilms by Acidic Electrolyzed Water. Front. Microbiol..

[66] Zhang H., Tian Y., Kang M., Chen C., Song Y., Li H. (2019). Effects of Chlorination/Chlorine Dioxide Disinfection on Biofilm Bacterial Community and Corrosion Process in a Reclaimed Water Distribution System. Chemosphere.

[67] Salisbury A.-M., Percival S.L. (2019). The Efficacy of an Electrolysed Water Formulation on Biofilms. Advances in Microbiology, Infectious Diseases and Public Health.

[68] Gebendorfer K.M., Drazic A., Le Y., Gundlach J., Bepperling A., Kastenmüller A., Ganzinger K.A., Braun N., Franzmann T.M., Winter J. (2012). Identification of a Hypochlorite-Specific Transcription Factor from *Escherichia coli*. J. Biol. Chem..

[69] Goemans C.V., Collet J.-F. (2019). Stress-Induced Chaperones: A First Line of Defense against the Powerful Oxidant Hypochlorous Acid. F1000Research.

[70] Gray M.J., Wholey W.-Y., Jakob U. (2013). Bacterial Responses to Reactive Chlorine Species. Annu. Rev. Microbiol..

[71] Wholey W.-Y., Jakob U. (2012). Hsp33 Confers Bleach Resistance by Protecting Elongation Factor Tu against Oxidative Degradation in *Vibrio cholerae*. Mol. Microbiol..

[72] Wang Z., Fang Y., Zhi S., Simpson D.J., Gill A., McMullen L.M., Neumann N.F., Gänzle M.G. (2020). The Locus of Heat Resistance Confers Resistance to Chlorine and Other Oxidizing Chemicals in *Escherichia coli*. Appl. Environ. Microbiol..

[73] Tong C., Hu H., Chen G., Li Z., Li A., Zhang J. (2021). Disinfectant Resistance in Bacteria: Mechanisms, Spread, and Resolution Strategies. Environ. Res..

[74] da Cruz Nizer W.S., Inkovskiy V., Overhage J. (2020). Surviving Reactive Chlorine Stress: Responses of Gram-Negative Bacteria to Hypochlorous Acid. Microorganisms.

[75] Liu S., Zhang Z., Gu P., Yang K., Huang X., Li M., Miao H. (2023). Elucidating Applied Voltage on the Fate of Antibiotic Resistance Genes in Microbial Electrolysis Cell: Focusing on Its Transmission between Anolyte and Biofilm Microbes. Sci. Total Environ..

[76] Chang H.-Y., Gui C.-Y., Huang T.-C., Hung Y.-C., Chen T.-Y. (2023). Quantitative Proteomic Analysis on the Slightly Acidic Electrolyzed Water Triggered Viable but Non-Culturable *Listeria monocytogenes*. Int. J. Mol. Sci..

[77] Hu X., Wang X., Ren H., Li C., Zhang B., Shi R., Wang Y., Lu S., Li Y., Lu Q. (2024). Preliminary Study of the Characterization of the Viable but Noncultivable State of *Yersinia enterocolitica* Induced by Chloride and UV Irradiation. Microorganisms.

[78] Li L., Mendis N., Trigui H., Oliver J.D., Faucher S.P. (2014). The Importance of the Viable but Non-Culturable State in Human Bacterial Pathogens. Front. Microbiol..

[79] Chen S., Zeng J., Wang Y., Ye C., Zhu S., Feng L., Zhang S., Yu X. (2020). Modelling the Effect of Chlorination/Chloramination on Induction of Viable but Non-Culturable (VBNC) *Escherichia coli*. Environ. Technol..

[80] Casini B., Baggiani A., Totaro M., Mansi A., Costa A.L., Aquino F., Miccoli M., Valentini P., Bruschi F., Lopalco P.L. (2018). Detection of Viable but Non-Culturable *Legionella* in Hospital Water Network Following Monochloramine Disinfection. J. Hosp. Infect..

[81] Ampiaw R.E., Yaqub M., Lee W. (2021). Electrolyzed Water as a Disinfectant: A Systematic Review of Factors Affecting the Production and Efficiency of Hypochlorous Acid. J. Water Process Eng..

[82] Iram A., Wang X., Demirci A. (2021). Electrolyzed Oxidizing Water and Its Applications as Sanitation and Cleaning Agent. Food Eng. Rev..

[83] Forster A.L.B., Wiskur S.L., Richardson S.D. (2025). Formation of Eight Classes of DBPs from Chlorine, Chloramine, and Ozone: Mechanisms and Formation Pathways. Environ. Sci. Technol..

[84] Stanford B.D., Pisarenko A.N., Snyder S.A., Gordon G. (2011). Perchlorate, Bromate, and Chlorate in Hypochlorite Solutions: Guidelines for Utilities. J. AWWA.

[85] Stanford B.D., Pisarenko A.N., Dryer D.J., Zeigler-Holady J.C., Gamage S., Quiñones O., Vanderford B.J., Dickenson E.R.V. (2013). Chlorate, Perchlorate, and Bromate in Onsite-Generated Hypochlorite Systems. J. AWWA.

[86] European Union (2020). Directive (EU) 2020/2184 of the European Parliament and of the Council of 16 December 2020 on the Quality of Water Intended for Human Consumption (Recast) (Text with EEA Relevance). Off. J. Eur. Union.

[87] Padhi R.K., Subramanian S., Satpathy K.K. (2019). Formation, Distribution, and Speciation of DBPs (THMs, HAAs, ClO2−, and ClO3) during Treatment of Different Source Water with Chlorine and Chlorine Dioxide. Chemosphere.

[88] Vertova A., Miani A., Lesma G., Rondinini S., Minguzzi A., Falciola L., Ortenzi M.A. (2019). Chlorine Dioxide Degradation Issues on Metal and Plastic Water Pipes Tested in Parallel in a Semi-Closed System. Int. J. Environ. Res. Public Health.

[89] Cullom A., Spencer M.S., Williams M.D., Falkinham J.O., Pruden A., Edwards M.A. (2023). Influence of Pipe Materials on In-Building Disinfection of *P. aeruginosa* and *A. baumannii* in Simulated Hot Water Plumbing. Water Res. X.

[90] Cullom A., Spencer M.S., Williams M.D., Falkinham J.O.I., Brown C., Edwards M.A., Pruden A. (2023). Premise Plumbing Pipe Materials and In-Building Disinfectants Shape the Potential for Proliferation of Pathogens and Antibiotic Resistance Genes. Environ. Sci. Technol..

[91] Ma X., Lytle D.A., Lee W.H. (2019). Microelectrode Investigation on the Corrosion Initiation at Lead–Brass Galvanic Interfaces in Chlorinated Drinking Water. Langmuir.

[92] Zhang H., Zhao L., Liu D., Wang J., Zhang X., Chen C. (2020). Early Period Corrosion and Scaling Characteristics of Ductile Iron Pipe for Ground Water Supply with Sodium Hypochlorite Disinfection. Water Res..

[93] Castagnetti D., Scirè Mammano G., Dragoni E. (2011). Effect of Chlorinated Water on the Oxidative Resistance and the Mechanical Strength of Polyethylene Pipes. Polym. Test..

[94] Eng J., Sassi T., Steele T., Vitarelli G. (2011). The Effects of Chlorinated Water on Polyethylene Pipes. Plast. Eng..

